# Regulation of *TREM2* expression by transcription factor YY1 and its protective effect against Alzheimer’s disease

**DOI:** 10.1016/j.jbc.2023.104688

**Published:** 2023-04-11

**Authors:** Yanhui Lu, Xiaofeng Huang, Wenping Liang, Yu Li, Mengen Xing, Wenhao Pan, Yun Zhang, Zhe Wang, Weihong Song

**Affiliations:** 1 The National Clinical Research Center for Geriatric Disease, Xuanwu Hospital, Capital Medical University, Beijing, China; 2 Zhejiang Provincial Clinical Research Center for Mental Disorders, School of Mental Health and The Affiliated Wenzhou Kangning Hospital, Institute of Aging, Key Laboratory of Alzheimer’s Disease of Zhejiang Province, Wenzhou Medical University, Oujiang Laboratory (Zhejiang Lab for Regenerative Medicine, Vision and Brain Health), Wenzhou, Zhejiang, China

**Keywords:** TREM2, YY1, transcription, gene regulation, Alzheimer’s disease

## Abstract

*TREM2* encoding the transmembrane receptor protein TREM2 is a risk gene of Alzheimer’s disease (AD), and the impairment of TREM2 functions in microglia due to mutations in *TREM2* may significantly increase the risk of AD by promoting AD pathologies. However, how the expression of *TREM2* is regulated and the transcription factors required for *TREM2* expression are largely unknown. By luciferase assay, DNA pull-down, and *in silico* predictions, we identified Yin Yang 1(YY1) as a binding protein of the minimal promoter of the *TREM2* gene, and the binding was further confirmed by EMSA and DNA pull-down assay. shRNA-mediated *YY1* silencing significantly reduced the activity of the *TREM2* minimal promoter and TREM2 protein levels in the microglial cell line BV2 and the neuroblastoma Neuro2A. Furthermore, we found that the levels of TREM2 and YY1 were both downregulated in lipopolysaccharide-treated BV2 cells and in the brain of AD model mice. These results demonstrated that YY1 plays a crucial role in the regulation of *TREM2* expression. Our study suggests that microglial YY1 could be targeted to maintain *TREM2* expression for AD prevention and therapy.

Alzheimer’s disease (AD) is the most common neurodegenerative disease leading to dementia in the elderly. The extracellular neuritic plaque with the amyloid protein (Aβ) as the major component and intracellular fibrillary tangle formed by the aggregation of hyperphosphorylated tau protein are the characteristic neuropathologies of AD (1). While how neuritic plaque and fibrillary tangle deposit in the brain of AD patients is not clear, impaired clearance of toxic components by the innate immune cells could contribute to the pathology (2).

Triggering receptor expressed on myeloid cells (TREM2) is a type I transmembrane receptor mainly expressed in myeloid lineage including microglia in the brain. Upon its binding with extracellular ligands on the plasma membrane, TREM2 initiates downstream signaling cascades through its cytoplasmic binding protein TYROBP (or DAP12) and is as such involved in a variety of cellular functions (3, 4). TREM2 in microglia is required for the regulation of immune responses and phagocytosis that are closely related to AD pathogenesis (5, 6, 7, 8).

Case-control studies revealed several rare mutations in *TREM2* gene increase the risk of AD. The carriers of the best characterized p.Arg47His in *TREM2* are 2.83 times more prone to AD, although the association is only confirmed in European population (9, 10). Functional studies suggest that these *TREM2* gene variations cause loss-of-function of TREM2 protein, resulting in not only AD but also other disorders (11, 12). TREM2 can be cleaved by ADAM10 and ADAM17 in the extracellular/intraluminal domain to release the C terminally truncated soluble TREM2 (sTREM2) into the interstitial or cerebrospinal fluid in the brain (13). The increased sTREM2 in the cerebrospinal fluid of early stage of AD could be due to enhanced cleavage, which may reduce functional TREM2 at the cell surface (13, 14, 15, 16).

Given the genetic and functional studies indicate that compromised TREM2 functions are correlated to AD, insufficient TREM2 expression could be a potential cause of AD (17). *In vitro* studies in microglial cells demonstrated that the expression of TREM2 is decreased by proinflammatory agents such as TNFα, IL1β, IFNγ, and lipopolysaccharide (LPS) (18, 19). However, how TREM2 expression is regulated, especially in the context of AD, remains elusive.

In this study, we identified Yin Yang 1 (YY1) as a transcription factor required for *TREM2* expression. An evolutionarily conserved YY1 response element close to the transcription starting site (TSS) of *TREM2* is indispensable for YY1-mediated TREM2 expression. In microglia cell challenged with LPS and in brains of AD model mice, both TREM2 and YY1 were significantly decreased. Therefore, microglial YY1 could be targeted to maintain TREM2 expression for AD prevention and therapy.

## Results

### Identification of the minimal active region in *TREM2* promoter

**Figure 1:**
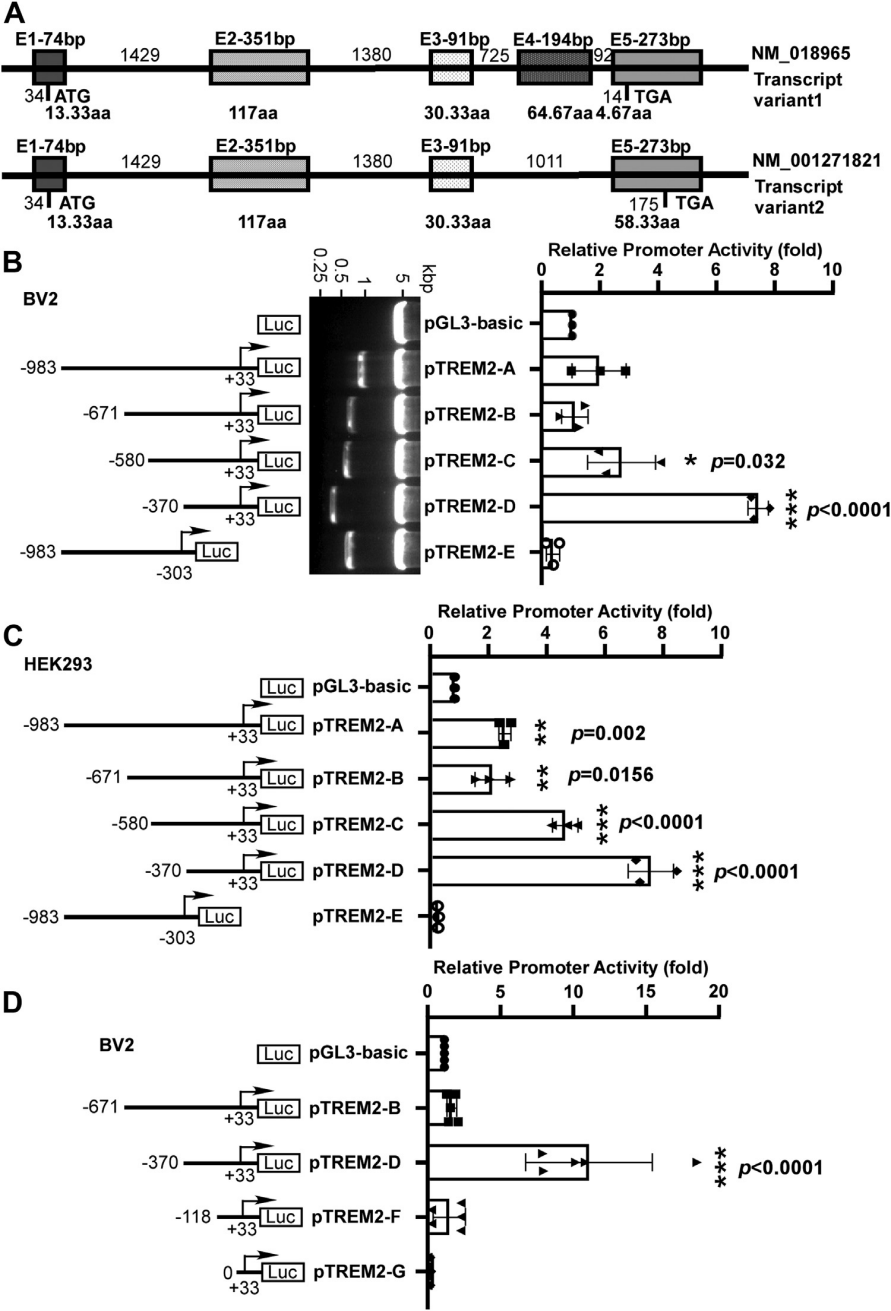
**Deletion analysis of the human *TREM2* gene promoter.***A*, the genomic features of human *TREM2* gene on chromosome 6p21.1. E represents exon. *TREM2* gene is comprised of five exons and contains two variants because of alternative splicing. ATG is the translation start codon and TGA is the translation stop codon. *B*, schematic illustration of human *TREM2* promoter deletion constructs in pGL3-Basic vector. The *arrow* represents the direction of transcription and the number demonstrates the start and ending point of each construct insert relative to the transcription start site. *TREM2* promoter deletion constructs were verified by restriction enzyme digestion, and the digested products were analyzed on 1.2 % agarose gel. The size of pGL3-Basic vector is 4.8 kb and inserts range from 337 bp to 1016 bp. The inserts were further confirmed by sequencing. The series of deletion constructs were cotransfected with pCMV-RLuc into BV2 cells. The cell lysates were harvested 24 h after transfection, and the luciferase activity was measured with a luminometer. The *TREM2* promoter luciferase activity was normalized by pCMV-Luc luciferase activity for transfection efficiency and expressed in folds in comparison with the luciferase activity of pGL3-Basic vector. *C*, the series of deletion constructs were cotransfected with pCMV-Luc into HEK293 cells and measured by luminometer. *D*, the shorter deletion constructs were cotransfected with pCMV-Luc into BV2 cells and measured by luminometer. The values represent the means±SD. ∗*p* < 0.05 by analysis of one-way ANOVA followed. HEK293, human embryonic kidney 293.

Human *TREM2* gene transcript can be spliced into two variants: the variant 1 is 693 bp in length and consists of five exons, whereas variant 2 is 660 bp in length and lacks exon 4 with larger exon 5. The two variants differ only in the 3′ ends and share the identical TSS. To investigate the transcriptional activity of the human TREM2 gene promoter, we extracted human genomic DNA and cloned a 983-bp fragment upstream the TSS (site 0, and the start codon ATG is +34--+36) (Fig. 1*A*). This fragment, and a series of 5′ deletion fragments, were cloned into pGL3-Basic vector for luciferase reporter assay. As in the brain, TREM2 is highly expressed in microglia, we first transfected the plasmids into the microglial cell line BV2 and cotransfected the plasmid pCMV-RLuc to express *Renilla* luciferase under the strong ubiquitous promoter CMV as an internal control (Fig. 1*B*). The plasmid pTREM2-A and pTREM2-B, containing −983∼+33 and −671∼+33, respectively, displayed a similar promoter activity compared to pGL3-Basic. The promoter activity of pTREM2-C containing −580∼+33 had slight increase by 2.679 ± 0.452 folds compared with vector. Further 5′ truncation down to −370∼+33 (pTREM2-D) significantly elevated the promoter activity by 6.391 ± 1.167 folds compared with pGL3-Basic. Another fragment −983∼−303 (pTREM2-E) showed nearly zero promoter activity and the luciferase expression under this fragment is even lower than that in PGL3-Basic. Similar difference in the promoter activities of these fragments were also found in human embryonic kidney 293 (HEK293) cells (Fig. 1*C*). It appears that in the −983∼+33 region, there are both positive and negative regulatory elements, with the latter within −580∼−370 bp region.

To further narrow down the core promoter region, additional 5′ deletion fragments −118∼+33 and 0∼+33 were cloned into PGL3-Basic to generate pTREM2-F and pTREM2-G. Both pTREM2-F and pTREM2-G had little promoter activity. These data suggested that there is a strong cis-acting element between −370 and −118 that spiked luciferase expression and could be crucial for *TREM2* expression (Fig. 1*D*).

### The *TREM2* gene promoter contains an YY1-binding site

**Figure 2:**
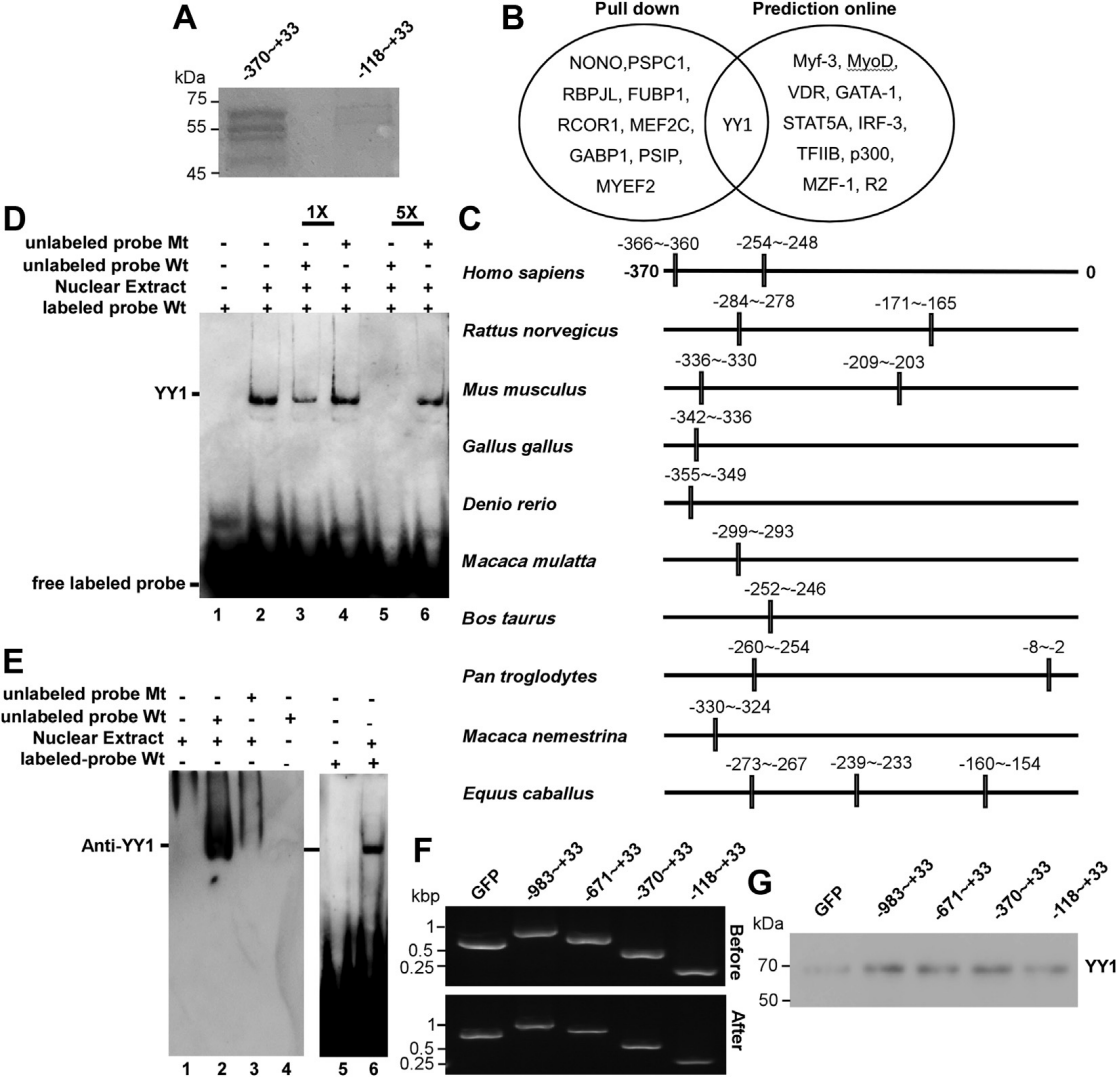
**The binding of *TREM2* gene promoter with transcription factor, YY1.***A*, the gel of pull-down assay for the −370∼−118 region of *TREM2* promoter–binding protein. *B*, the venn diagram of potential transcription factors predicted by two different methods. The left cluster demonstrates proteins binding with −370∼+33 fragment but not −118∼+33 by mass spectrum. The right cluster indicates potential transcription factors predicted by online tool PROMO. The intersection set represents that YY1 was predicted as potential transcription factors by both mass spectrum and online tool. *C*, the putative YY1 response elements in 5′ flanking region of *TREM2* gene in eight different species. *D*, electrophoretic mobility shift assays (EMSAs) for the binding of *TREM2* gene promoter and transcription factor, YY1. Nuclear extract was isolated from BV2 cells, and YY1 hot probe was labeled with biotin. Lane 1 is the double strands oligonucleotides probe labeled by biotin without nuclear protein extract, illustrating there was no nonspecific band if only containing YY1 probe. Lane 2 indicates a shifted DNA–protein complex band formed by incubation of BV2 nuclear extract and labeled YY1 probe. Lane 3 to 6 denotes cold competition by the addition of unlabeled 1-fold WT YY1 probe (line 3), 1× mutant YY1 probe (line 4), 5× WT YY1 probe (line 5), and 5× mutant YY1 probe (line 6), respectively. *E*, anti-YY1 antibody specifically recognizes YY1 protein in the complex of unlabeled probe and nuclear extract. Lane 1 contains only nuclear extract. Lane 2, the unlabeled probe incubated with nuclear extracts. Lane 3 consists of both nuclear extract and mutant cold probe. Lane 4 only contains YY1 hot probe. Lane 5 and lane 6 were the EMSA result showing that the band in lane 2 migrated at the same position as the upshifted band in EMSA. n = 4 independent repeats. *F*, before and after incubation of probes and streptavidin magnetic beads, the amounts of probes were measured by agarose gel to verify the binding efficiency, and the beads were used for YY1 pull-down from nuclear extract as in (*G*). *G*, pull-down assay for the binding of YY1 and *TREM2* gene promoter. Different DNA fragments 5′ to the transcriptional starting site of *TREM2* were used to pull-down YY1 in nuclear extracts. cDNA coding for GFP was used as a negative control DNA. The precipitated proteins were blotted for YY1. YY1, Yin-Yang 1.

To identify transcription factors binding to the −370∼−118 region of *TREM2* promoter, we first performed DNA pull-down assay using PCR-produced and biotinylated bait fragments −370∼+33 and −118∼+33. Proteins pulled down from BV2 nuclear extracts and stained by Coomassie blue were excised from the gel and subjected to mass spectrometry analysis (Fig. 2*A*). A number of transcription factors including YY1 were found to specifically bind with −370∼+33 but not with −118∼+33 (Fig. 2*B*). To further narrow down the candidate transcription factors crucial for the promoter activity of −370∼+33, a computational transcription factor prediction analysis using online tool PROMO was performed (Fig. 2*B*). By comparing the results from DNA pull-down assay and the online prediction, YY1 was the only putative transcription factor for human *TREM2* gene by both methods. Moreover, by online prediction, we also identified putative YY1 response elements in the similar region upstream of *TREM2* TSS in eight lower species (Fig. 2*C*). Hence, potential YY1-regulated *TREM2* expression, if true, is highly conserved during evolution.

To confirm the specific binding of YY1 to the predicted binding site, electrophoretic mobility shift assay (EMSA) was conducted. According to the computational database analysis, the YY1 cis-acting element locates at −254∼−248, with the sequence CCATCTG. A 25-bp double-stranded oligonucleotides probe containing YY1-binding element in TREM2 gene was synthesized and double labeled with biotin. An upshift band upon the incubation with BV2 nuclear extract was observed (Fig. 2*D* line 3). The intensity of the upshifted band was partially decreased by the addition of 1× unlabeled WT oligonucleotide probe (Fig. 2*D* line 4) and was totally abolished by the addition of 5× WT unlabeled probe (Fig. 2*D* line 6). Mutant probes with the putative YY1-binding site changed from CCATCTG into GGGGCTG did not affect the upshifted band at either concentration (Fig. 2*D* lanes five and 7).

To confirm the upshifted band in EMSA did contain YY1 and probe complex, we attempted super shift assay to further shift the band using anti-YY1 antibody. However, we failed to find antibodies available for applications such as immunoprecipitation or EMSA. Therefore, we used an alternative method to combine EMSA and Western blot. After the gel separation as in EMSA, we transferred proteins on the gel onto nitrocellulose membranes and performed Western blot using anti-YY1 antibody. A YY1 band was detected at the same site as the upshifted band in EMSA when the WT probe was incubated with BV2 nuclear extract. Incubation of the mutant probe with the nuclear extract or the nuclear extract alone did not yield such a band (Fig. 2*E*). Hence, YY1 does form a complex with the probe containing the putative YY1 response element.

Since the longer fragments extending from the 5′ end of −370∼+33 showed much lower promoter activity than −370∼+33, we wondered if they could form secondary or higher structures to impair the binding of YY1 with the response element and therefore performed DNA pull-down assay to compare the binding of these fragments with YY1 in nuclear extracts. Before and after incubation, the quantity of probes was measured by agarose gels. The results showed that there were excessive probes remained in the flow through, which indicated that all the beads were saturated by equal amounts of probes (Fig. 2*F*). All the longer fragments but not the cDNA of GFP or the fragment −118∼+33 pulled down similar amounts of YY1 (Fig. 2*G*). Thus, there could be other *cis*-elements in these longer fragments to suppress *TREM2* expression.

### YY1 is required for *TREM2* promoter activity and TREM2 protein expression

**Figure 3:**
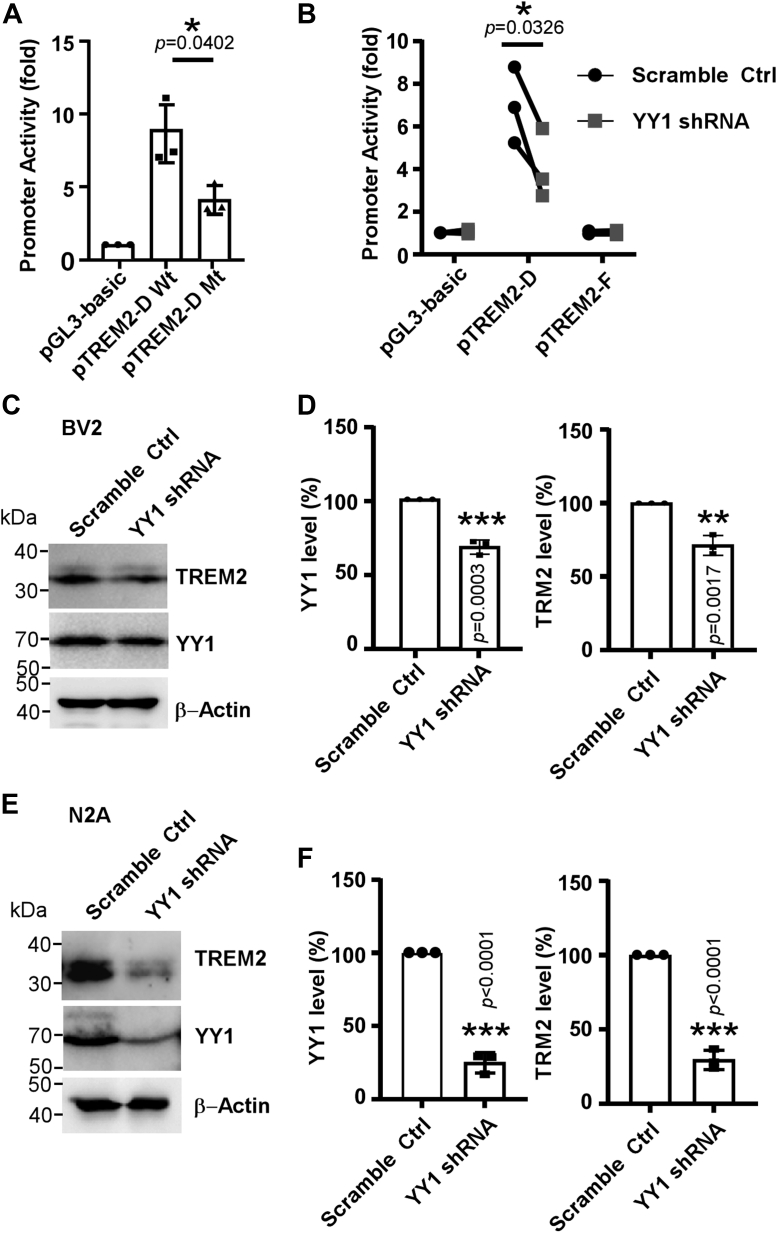
**YY1 regulates the human *TREM2* gene promoter activity.***A*, the plasmids of pTREM2-D (wt) and the mutant pTREM2-D (mt) containing the same mutations as those in the mutant probe for EMSA were cotransfected with pCMV-RLuc into BV2 cells. Twenty-four hours after transfection, the luciferase activity was assessed by luminometer and expressed as change fold in comparison with pGL3-Basic vector. The values represent means ± SD, n = 3 independent repeats, ∗*p* < 0.05 *versus* pGL3-Basic vector by one-way ANOVA. *B*, the effect of *YY1* silencing by shRNA on *TREM2* gene promoter activity. shRNAs were transfected into neuro2A cells 24 h before the transfection of luciferase reporter plasmids. The luciferase activity was measured by luminometer and expressed as change fold in comparison with pGL3-Basic vector. The values represent means ± SD, n = 3 independent repeats, ∗*p* < 0.05 *versus* pGL3-Basic vector by two-way ANOVA. *C*, the effect of *YY1* silencing on the expression of TREM2 protein in BV2. Forty-eight hours after shRNA transfection, the cells were lysed and blotted for YY1 and TREM2. *D*, the protein levels of YY1 and TREM2 was quantified in comparison with control. *E*, *YY1* silencing in neuro2a decreased endogenous TREM2 protein. Forty-eight hours post shRNA transfection, the cells were lysed and blotted for YY1 and TREM2. *F*, the protein levels of YY1 and TREM2 in neuro2a (N2A) was quantified in comparison with control. The values represent means ± SD, n = 3 independent repeats, ∗*p* < 0.05 *versus* control by Student’s *t* test. YY1, Yin-Yang 1; EMSA, electrophoretic mobility shift assay.

To confirm if YY1 and the putative response element are functionally involved in the transcriptional regulation of *TREM2* gene and TREM2 expression in cells, we first generated a mutant pTREM2-D plasmid that contains the same mutations as those in the mutant probe for EMSA (pTREM2-D Mt). The mutation remarkably reduced pTREM2-D’s promoter activity by 53.021 ± 16.86% (Fig. 3*A*). Additionally, shRNA-mediated *YY1* silencing suppressed the promoter activity of pTREM2-D by 45.43 ± 14.55% (Fig. 3*B*), suggesting that pTREM2-D contains YY1 response element.

In BV2 cells, shRNA-mediated *YY1* silencing only mildly reduced endogenous YY1 protein by about 30%, which was associated with simultaneous reduction of TREM2 at a similar percentage (Fig. 3, *C* and *D*). In neuro2A cell, a neuroblastoma cell line expressing endogenous TREM2 (20), the same shRNA against *YY1* caused more than 70% decrease of YY1 and TREM2 proteins (Fig. 3, *E* and *F*). Together, YY1 is indispensable for *TREM2* expression.

### Decreased YY1 and TREM2 expression by LPS and in AD transgenic mice

**Figure 4:**
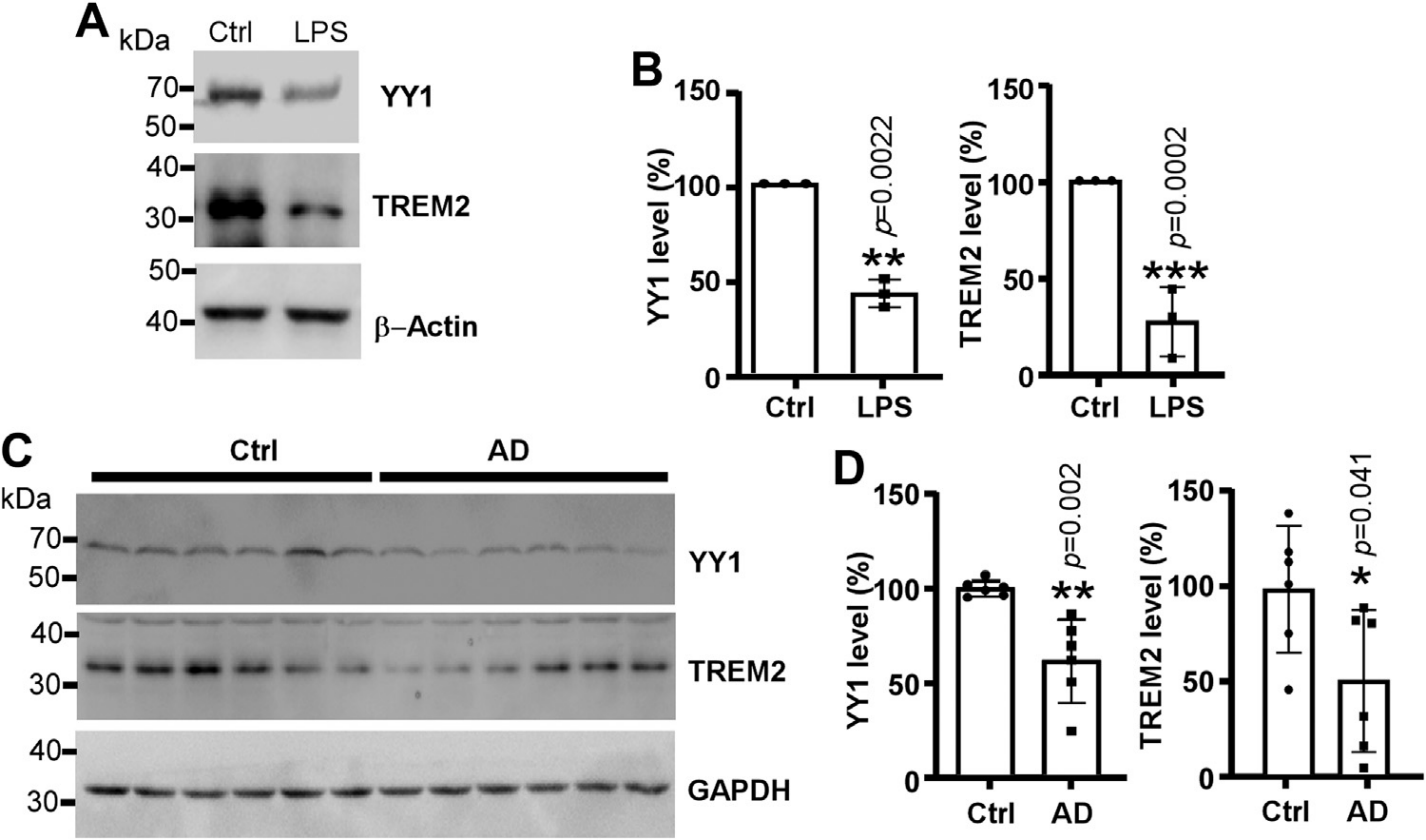
**Both YY1 and TREM2 respond to inflammation and AD pathologies.***A*, BV2 cells were treated with 2 μg/ml LPS for overnight. TREM2 and YY1 were detected by immunoblotting. *B*, the protein level of TREM2 and YY1 was quantified in comparison with control. The values represent means ± SD, n= 3 independent repeats, ∗*p* < 0.05 *versus* control by Student’s *t* test. *C* and *D*, the protein level of TREM2 and YY1 in WT mice and age matched AD mice were determined and quantified. The values represent means ± SD, n= 6 mice in each group, ∗*p* < 0.05 *versus* control by Student’s t test. AD, Alzheimer's disease; LPS, lipopolysaccharide; YY1, Yin-Yang 1.

It has been well established that TREM2 in microglia is downregulated by the inflammation eliciting agent LPS (18, 21). We found that YY1 was also decreased by LPS at 2 μg/ml in BV2 cells (Fig. 4, *A* and *B*). Hence, it is possible that the reduction of TREM2 could be a consequence of YY1 suppression. We further tested if the overexpression of YY1 could rescue the decrease of TREM2 under the condition of LPS, however, the overexpression of functional YY1 in BV2, especially in the context of LPS, was extremely weak, and the increase of TREM2 protein, if any, was only marginal (data not shown).

YY1 may also be involved in TREM2 expression *in vivo*. Compared with age- and gender-matched WT mice, both YY1 and TREM2 in the *APP/PS45* AD model mice (22) were decreased by 38.84 ± 9.339% and 49.25 ± 21.0%, respectively (Fig. 4, *C* and *D*).

## Discussion

Deficiency in *TREM2* significantly increases the risk of AD. In microglia, the major cell type in the brain to express TREM2, TREM2 participates in multiple cellular functions such as mediating the phagocytosis of Aβ, suppressing the inflammatory, and orchestrating lipid metabolism (23). All of these functions of microglia are compromised in AD (4). The mutations/SNPs in *TREM2* correlated to AD may blunt some of these functions and as such increase the risk of AD (24, 25, 26). In nonmutation/SNP carriers, *TREM2* may be involved in AD pathogenesis through altered expression (5, 27), and the decreased TREM2 expression compromises the functions of TREM2 (28). However, how TREM2 expression is regulated and the transcription factors required for TREM2 expression are largely unknown.

To identify the minimal promoter region of *TREM2* and the transcription factors necessary for *TREM2* expression, we generated a series of fragments upstream TSS of *TREM2* gene. It was interesting to note that although the longest fragment (−983∼+33) we tested showed little promoter activity, when it was truncated down to −370∼+33, the promoter activity spiked up. Further truncation of −370∼+33 to −118∼+33 abolished promoter activity. Together, these data indicated that there could be repressing cis-element between −580∼−370 and activating cis-element between −370∼−118. We also noticed that the fragments pTREM2-E (0∼−33) and G (−983∼−303) in PGL3-Basic displayed even lower promoter activity than the empty PGL3-Basic vector. One possible explanation is that these fragments replaced the multiple cloning sites of PGL3-Basic that may have a weak promoter activity. If this is the case, the promoter activity of −370∼+33 could have been under estimated.

YY1 is ubiquitously expressed in mammalian cells and serves as both transcription activator and repressor depending on its modifications, cofactors, chromatin structures, and target genes (29, 30). Some studies suggested that YY1 in neurons may increase Aβ by regulating the expression of proteins directly and indirectly involved in Aβ production (31, 32, 33). Biopsy examinations indicated that in the hippocampus and temporal cortex of AD patients, YY1 decreases and the proteolytic fragments of YY1 increases. Moreover, YY1 was also found to be reduced in the brains of patients with other neurodegenerative disease (34). We found that YY1 in BV2 is decreased by LPS, a condition to simulate neuroinflammation that is common for almost all neurodegenerative diseases. However, LPS was shown to increase YY1 activity in B cell in the periphery (35). Our results suggested that YY1 may directly promote *TREM2* expression in microglia, which in turn enhances the clearance of Aβ, suppresses the immune responses, and maintains the cell homeostasis of microglia. Interestingly, YY1 was also reported to indirectly upregulate TREM2 through miRNA (36). Thus, to maintain microglial *TREM2* expression by enhancing YY1 activity could be a potential strategy for AD prevention and diagnosis.

## Experimental procedures

### Plasmids construction

The 5′ flanking region of *TREM2* gene was generated by PCR amplification of HEK293 cells’ genomic DNA. The deletion fragments were amplified by PCR with specific primers and inserted into pGL3-Basic vectors which were digested by XhoI and HindIII. The primers sequence used for pTREM2-A, pTREM2-B, pTREM2-C, pTREM2-D, pTREM2-E, pTREM2-F, and pTREM2-G were listed as the following: −983fXHoI: 5′-gccCTCGAGcaccatgggaacctgtacgtgtag, −671fXhoI: 5′- gccCTCGAGgttgaatgctgtgtgtcaggc, −580fXHoI: 5′-cccCTCGAGcccactgtatagatcagggaac, −370fXHoI: 5′-gccCTCGAGcagaagatggcgggcattg, −118fXHoI: 5′-gccCTCGAGagaccccagtcctgactattgc, −303rHindIII: 5′-gccAAGCTTcagtttccttgcagagcctag, +33rHindIII: 5′-gccAAGCTTccacccttccccagccaag. A deletion mutation of the putative YY1-binding site based on pTREM2-D was constructed with primers listed as the following: fMutation: gggccttaccagcccca∧tgggggccaccctggctgg, rMutation: ccagccagggtggccccca∧tggggctggtaaggccc.

### Cell culture, transfection, and luciferase reporter assay

HEK293 cells and BV2 cells were purchased from the American Tissue Culture Collection and were cultured in Dulbecco’s modified Eagle’s medium containing 10% fetal bovine serum, 1 mM sodium pyruvate, 2 mM L-glutamine (servicebio) at 37 °C in a 5% CO2 and 95% air in an incubator. HEK293 cells and BV2 cells were cotransfected with 500 ng TREM2 promoter constructs and 1 ng pCMV-RLuc per well of 24-well plate with lipo8000 (Beyotime) for luciferase assay. Luciferase assay was performed according to technical manual of Dual-Luciferase Reporter Assay System (Promega). Cell lysates were harvested and lysed with 100 μl passive lysis buffer per well after 24 hours transfection. *Firefly* luciferase activities and *Renilla* luciferase activities were measured sequentially by the Dual-Luciferase Reporter Assay System (Promega). The *firefly* luciferase activity was normalized with *Renilla* luciferase activity and represented as relative folds in comparison with pGL3-Basic vector activity. The *YY1* targeting sequence of the shRNA (5′ GTGGTTGAAGAGCAGATCATTTTCAAGAGAAATGATCTGCTCTTCAACCACTTTTTT 3′) was cloned into pAV-U6-shRNA-CMV-intron-GFP vector for the expression under U6 promoter.

### DNA pull-down and mass spectrum

The biotin-labeled probes used in pull-down assays correspond to −983∼+33, −671∼+33, −370∼+33, and −118∼+33 of *TREM2* gene 5′ flanking region and genomic *GFP*, respectively. The probes were rotated with streptavidin magnetic beads (P2151, Beyotime) for 3h at 4 °C in binding & washing buffer (10 mM Tris–HCl (pH 7.5), 1 mM EDTA, 2M NaCl, 0.01%-0.1% Tween-20). Before and after incubation, the quantity of probes was measured by agarose gels. And then, the DNA–beads complex was washed three times and incubated with BV2 nuclear extracts in rotation overnight at 4 °C. Following with incubation, the protein–DNA–beads complex was washed three times and lysed by boiling in SDS loading buffer. The samples were then resolved in 10% SDS-PAGE system and analyzed by immunoblotting.

### Electrophoretic mobility shift assay

The EMSA for YY1 was performed using the Lightshift Chemiluminescent EMSA kit (Pierce) according to the manufacturer’s instruments. BV2 cells were harvested and subsequently lysed in a series of hypotonic buffers for nuclear extraction protein. Probe oligonucleotides were labeled with or without biotin and annealed to produce double-strand oligonucleotide probes. The probes were incubated with or without nuclear extract at 22 °C for 20 min in the EMSA-binding buffer. For the competition assay, nuclear extract was incubated with 1× or 5× concentration of unlabeled competition oligonucleotides as well as labeled probes.

The sequences of the oligonucleotides were as follows: YY1 probe-WT: 5′-CCAGCCCCAACCATCTGGGGGCCAC-3′, YY1 probe-mutation: 5′-CCAGCCCCAAGGGGCTGGGGGCCAC-3′

### Immunoblotting

Cells were harvested and lysed by sample buffer followed by Ultrasonic Cell Crusher. Cells lysate were resolved on 8% Tris-glycine or 16% Tris-tricine gels (Bio-Rad), following with transfer into nitrocellulose membrane (Millipore). The nitrocellulose membrane was blocked with 5% nonfat for 1h at room temperature and then blotted with primary antibody including TREM2 (E7P8J, Cell Signaling Technology) and YY1 (ET1605-40, HUABIO). After washing three times with PBST, the membranes were incubated with HRP-conjugated goat anti-mouse or anti-rabbit antibodies at room temperature for 2 h. After washing three times with PBST again, the membrane was visualized with ECL (Tanon 5800) and quantified by the Quantity one software (https://www.bio-rad.com). For the EMSA/Western blot assay, the nuclear extract and probe mixture after reaction was separated on a native gel as in conventional EMSA, and the gel was immersed in SDS-PAGE running buffer for 30 min to allow the incorporation of SDS into the proteins and denaturation of proteins. The proteins on the gel were then electrotransferred onto nitrocellulose membrane and blotted as in the conventional Western blot.

### Mouse brain tissues

APP/PS45 at the age of 3∼4 months, the time when neuritic plaques are abundant in the cortex and hippocampus, were derived from the crossing of APP23 mice and PS45 mice (22), and the brains were lysed in RIPA buffer. All animal experiments were approved by the IACUC (Institutional Animal Care and Use Committee) at the Wenzhou Medical University.

### Statistics

Three or more independent experiments were performed. All results are presented as mean ± SD and were analyzed by one-way ANOVA or two-tailed Student’s *t* test. *p* < 0.05 was considered as statistically significant.

## Data availability

All data are contained within the article.

## Conflict of interest

The authors declare that they have no conflicts of interest with the contents of this article.
